# Strangulated Hernia Reduced by Taxis after Puncturing the Intestines

**Published:** 1872-12

**Authors:** 


### STRANGULATED HERNIA REDUCED BY TAXIS AFTER PUNCTURING THE INTESTINE.

M. Demarquay advocated, before the Academie de Medicine, on May 21, 1872, the puncturing the gut with a small trocar in certain cases of hernia, and letting out the liquid and gas by means of Potain's aspirator. His operation on a hernial tumor, thirty-six hours old, after unsuccessful attempts at reduction by taxis and treating with ice, was a marked success. The patient's features had undergone great change, and fever was set up. A congenital, elongated, voluminous, inguinal hernia was found to exist. Having never succeded in curing this description of hernia by operation, he resolved on puncture. About 128 grammes of intestinal liquid were drawn out, besides considerable gas. The tumor collapsed completely—trocar was withdrawn, and some minutes were allowed to elapse, to see if the tumor would again fill. No renewal of the tumefaction took place, and very slight pressure returned the tumor into the abdominal cavity. Quiet, low diet, and small doses of opium, completed the cure. No ill consequences.

M. Demarquay proposes to apply this new mode of treatment.

1. In all congenital hernias, and to recent hernias which become strangulated at the time of their formation. 2. To old hernias which were quite reducible a few days prior to strangulation, and in large umbilical hernias that have become recently strangulated. Aspiration should be employed at an early period, when one can be well nigh certain of returning into the abdomen the intestine in an unaltered state and capable of resuming its functions.—*Chicago Medical Journal.*

